# Versorgungswirklichkeit ausgewählter kinderurologischer Eingriffe in Deutschland von 2006 bis 2019

**DOI:** 10.1007/s00120-021-01636-z

**Published:** 2021-09-15

**Authors:** Markus Maier, Anne-Karoline Ebert, Martin Baunacke, Christer Groeben, Nicole Eisenmenger, Christian Thomas, Johannes Huber

**Affiliations:** 1 Klinik für Urologie und Kinderurologie, Universitätsklinikum Marburg, Philipps Universität Marburg, Marburg, Deutschland; 2 Klinik für Urologie und Kinderurologie, Universitätsklinikum Ulm, Ulm, Deutschland; 3 Klinik und Poliklinik für Urologie, Universitätsklinikum Carl Gustav Carus, Technische Universität Dresden, Fetscherstraße 74, 01307 Dresden, Deutschland; 4 Reimbursement Institute, Hürth, Deutschland

**Keywords:** Interdisziplinarität, Hypospadie, Orchidopexie, Vesikoureteraler Reflux, Versorgungsforschung, Interdisciplinarity, Hypospadia, Orchidopexy, Vesicoureteral reflux, Health services research

## Abstract

**Hintergrund:**

Die konservative und chirurgische Behandlung von Kindern ist in der urologischen Facharztweiterbildung fest verankert und stellt eine Kernkompetenz der urologischen Versorgung dar. Berufspolitisch wird seit vielen Jahren ein zunehmender Verlust dieses Schwerpunkts befürchtet. Ziel dieser Studie ist es, reale Fallzahlen und eine mögliche Dynamik in der Verteilung kinderurologischer Eingriffe auf die Fachabteilungen für Urologie und Kinderchirurgie in Deutschland zu prüfen.

**Material und Methoden:**

Als Index-Eingriffe definierten wir Operationen des Hodenhochstands, der Hypospadie und des vesikoureteralen Refluxes (VUR). Mithilfe der Software reimbursement.INFO (RI Innovation GmbH, Hürth) werteten wir die öffentlich zugänglichen Qualitätsberichtsdaten der deutschen Krankenhäuser von 2006 bis 2019 aus und wiesen sie den entsprechenden Fachrichtungen zu.

**Ergebnisse:**

Die Orchidopexie erfolgt häufiger in der Urologie, wohingegen die Hypospadiekorrektur und die operative Therapie des VUR häufiger in der Kinderchirurgie durchgeführt werden. Anteilig zeigte sich für die Orchidopexie und die operative Refluxtherapie keine relevante Verschiebung zwischen urologischen und kinderchirurgischen Kliniken im Untersuchungszeitraum. Bei den Hypospadiekorrekturen nimmt der Anteil der Operationen in kinderchirurgischen Einheiten zu (*p* < 0,0001). In der Kinderchirurgie erfolgen 84–93 % der analysierten Eingriffe in High-volume-Abteilungen während dieser Anteil in der Urologie bei 56–73 % liegt. Insbesondere die operative Refluxtherapie in der Urologie erfolgt zu einem hohen Anteil als Gelegenheitseingriff (30 % „very low volume“).

**Schlussfolgerung:**

Die Qualitätsberichtsdaten ermöglichen die Erfassung der Fallzahlen und die Analyse der Verteilung zwischen Urologie und Kinderchirurgie in Deutschland. Dabei ist für die Hypospadiekorrektur eine relevante Verschiebung in Richtung der Kinderchirurgie zu beobachten. Die Ursachen und möglichen berufspolitischen Konsequenzen dieser ersten Erhebung sind komplex und bedürfen weiterer Analysen.

## Abstract (en)

**Background:**

The conservative and surgical treatment of children is a fundamental pillar of the urological specialist training program and represents one of the core competencies within urological healthcare. The loss of this expertise has been a reoccurring topic within urologic occupational policy. The aim of this study is to analyse actual case numbers and to compare the distribution and dynamics of pediatric urologic surgeries between the specialist departments of urology and pediatric surgery in Germany.

**Materials and methods:**

We defined the surgical treatments of maldecensus testis, hypospadias, and vesicoureteral reflux (VUR) as index interventions. Using the tool reimbursement.INFO (RI Innovation GmbH, Hürth, Germany) we analysed publicly available quality report data of German hospitals between 2006 and 2019.

**Results:**

While orchidopexy was more commonly performed in the field of urology, the correction of hypospadias and the surgical treatment of VUR showed higher case numbers in the field of pediatric surgery. Proportionally, there was no relevant shift between urologic and pediatric surgical clinics for orchidopexy and surgical VUR therapy during the study period. For hypospadias corrections, the proportion of surgeries performed in pediatric surgical units is increasing (*p* < 0.0001). In pediatric surgery 84–93% of the analyzed procedures are performed in high-volume units, while this proportion is 56–73% in urology. In particular, a high proportion of VUR therapy in urology is performed as an occasional procedure (30% very low volume).

**Conclusions:**

The quality report data enable the compilation of case numbers and the analysis of the distribution between urology and pediatric surgery in Germany. Merely the correction of hypospadias has shown a relevant shift towards pediatric surgery. The causes and possible consequences for professional policy of this preliminary investigation are complex and require further analysis.

## Hintergrund und Fragestellung

Die konservative und chirurgische Behandlung urologischer Krankheitsbilder im Kindesalter ist in der urologischen Facharztweiterbildung fest verankert und stellt eine wichtige Kompetenz der urologischen Versorgung dar. Jedoch wird seit vielen Jahren ein zunehmender Verlust dieses Schwerpunkts befürchtet.

Endsprechend berücksichtigte die Deutsche Gesellschaft für Urologie e. V. (DGU) die Kinderurologie auch in ihrem Konzeptpapier „Zukunftsoffensive Urologie 2025“ [1]. Dabei wird der Wunsch nach einer weiterhin qualitativ hochwertigen Versorgung der Kinder in der Urologie klar formuliert. Die Mitglieder des Arbeitskreises Kinder- und Jugendurologie haben Empfehlungen formuliert, welche Krankheitsbilder flächendeckend behandelt werden sollten, z. B. die operative Versorgung des Hodenhochstands oder der Phimose, und für welche eine spezielle Expertise in wenigen definierten Zentren vorgehalten werden muss. Hierzu zählen die Hypospadiekorrektur, die Therapie des vesikoureteralen Reflux (VUR) und die Versorgung seltener kinderurologischer Anomalien wie den „differences in sex development“ (DSD), dem Blasenekstrophie-Epispadie-Komplex (BEEK) oder der Spina bifida. Vor diesem Hintergrund wurde folgerichtig als Konsens zwischen der DGU und der Deutschen Gesellschaft für Kinderchirurgie e. V. (DGKCH) die standardisierte Zusatzweiterbildung „Spezielle Kinder- und Jugendurologie“ etabliert, die nach der Facharztanerkennung für Urologie oder Kinderchirurgie über 18 Monate nur in spezialisierten Zentren mit kinderurologischer Expertise erworben werden kann. Das an Versorgungs- und Ausbildungsqualität orientierte Curriculum der „Speziellen Kinder- und Jugendurologie“ umfasst Kenntnisse und Fähigkeiten und legt auch zu erbringende Operationszahlen fest. Wissenschaftliche Fallzahlerhebungen operativer kinderurologischer Eingriffe in Deutschland standen bis dato nicht zur Verfügung.

Ziel unserer Studie ist es, in Deutschland für den Zeitraum von 2006 bis 2019 die Häufigkeit kinderurologischer Eingriffe sowie den jeweiligen Anteil der Fachabteilungen für Urologie (URO) und Kinderchirurgie (KCH) daran zu ermitteln. Als Indexeingriffe wählten wir die operative Behandlung des Hodenhochstands, der Hypospadie und des VUR.

## Material und Methoden

Um die Entwicklung ausgewählter kinderurologischer ambulanter und stationärer Eingriffe durch die Abteilungen für Urologie und für Kinderchirurgie in Deutschland zu analysieren, wurden die Qualitätsberichte deutscher Krankenhäuser verwendet. Seit 2005 sind deutsche Krankenhäuser gesetzlich verpflichtet, in Qualitätsberichten detaillierte Informationen über ihr Tätigkeitsspektrum und Strukturen zu geben. Die Datengüte der Qualitätsberichte der Krankenhäuser hängt dabei von der Dokumentation des jeweiligen Krankenhauses ab. Um die Daten der Krankenhäuser für die Jahre 2006 bis 2019 zu extrahieren, wurde das Analysetool reimbursement.INFO (RI Innovation GmbH, Hürth) verwendet. Aus Datenschutzgründen werden in Qualitätsberichten Eingriffe, die nur 1‑ bis 3‑mal pro Jahr im Krankenhaus durchgeführt werden, anonymisiert und für die vorliegende Arbeit mit der Fallzahl 1 dargestellt. Die Daten zu Alter und Geschlecht sind vom Statistischen Bundesamt verfügbar und beinhalten nur Informationen zu stationären Krankenhausfällen.

Folgende Operationen- und Prozedurenschlüssel (OPS-Codes) wurden zugrunde gelegt:

1. Orchidopexie: 5‑624, 5‑625 und 5‑626
2. Hypospadiekorrektur: 5‑645, 5‑645.0, 5‑645.1, 5‑645.2, 5‑645.20, 5‑645.21, 5‑645.22, 5‑645.23, 5‑645.2x, 5‑645.3, 5‑645.x, 5‑645.y. Darüber hinaus existieren Codes zu Korrektureingriffen der Urethra und des Penis, welche die Hypospadiekorrektur als Indikation explizit ausschließen. Diese Codes 5-584 und 5‑643 wurden nicht aufgenommen, obwohl sie teilweise für Rezidiveingriffe oder die Behebung von Komplikationen nach Hypospadiekorrektur genutzt werden. Eine sinnvolle Berücksichtigung ist hier perspektivisch nur durch die Kombination mit der Haupt- oder Nebendiagnose „Hypospadie“ (ICD-10-GM Q54) möglich.
3. Therapie des VUR: 5‑568.9, 5‑568.9x, 5‑568.90, 5‑568.91, 5‑568.8, 5‑568.80, 5‑568.81, 5‑568.8x, 5‑569.6, 5‑569.60, 5‑569.61, 5‑569.62 und 5‑569.6x. Der Code 5-568.d0 wurde nicht mit aufgenommen, da nur 24 % dieser Fälle Kinder sind; damit war dieser Code für die aktuelle Arbeit zu unspezifisch.

Der Anteil der PatientInnen < 20 Jahre in diesen OPS-Codes lag 2019 bei 84 % für die Orchidopexie, bei 94 % für die Hypospadiekorrektur und bei 69 % für die Therapie des VUR.

Die entsprechenden Fachabteilungen wurden durch den Fachabteilungsschlüssel (FAB) Urologie (FAB 2200) und Kinderchirurgie (FAB 1300 und FAB 1513) klassifiziert.

Alle anderen entlassenden Fachabteilungen wurden als „sonstige“ zusammengefasst, wobei hier die Pädiatrie die größte Gruppe ausmachte. Der Anteil an in der Pädiatrie abgerechneten Prozeduren zwischen 2006–2019 beträgt 8–12 % der Orchidopexien, 5–29 % der Hypospadiekorrekturen und 5–12 % der Refluxtherapien. Soweit möglich ordneten wir die aus der Pädiatrie (FAB 1000) entlassenen Fälle der Urologie oder der Kinderchirurgie zu, wenn dies anhand der verfügbaren Informationen über die Klinik möglich war.

Wir teilten die Leistungserbringer entsprechend ihrer Fallzahl im Jahr 2019 in Gruppen hinsichtlich der erbrachten Fallzahlstärke ein. Hierfür orientierten wir uns am durchschnittlichen jährlichen Fallaufkommen pro Abteilung und definierten „high-volume“ als eine über diesem Durchschnitt liegende Fallzahl. Damit lag die Fallzahlgrenze bei ≥ 25 Fällen/Jahr für die Orchidopexie und die Hypospadiekorrektur sowie bei ≥ 7 Fällen/Jahr für die Therapie des VUR. Von den verbleibenden Abteilungen grenzten wir eine Very-low-volume-Gruppe ab, die maximal eine Prozedur pro Quartal durchführte (< 5 Fälle/Jahr). Die Kartendarstellungen erfolgten mit der Software easymap© office (Lutum + Tappert DV-Beratung GmbH, Bonn).

Als Lage- und Streuungsmaße berichten wir den Mittelwert und den Interquartilsabstand (IQR). Zum Gruppenvergleich dienten der χ^2^- und der Mann-Whitney-U-Test. Lineare Regressionsanalysen analysierten Trends über die Zeit. Die Signifikanzgrenze definierten wir mit *p* < 0,05. Alle Berechnungen wurden mit IBM SPSS Statistics 27 (Armonk, NY, USA) durchgeführt.

## Ergebnisse

### Fallzahlentwicklung

**Figure 1:**
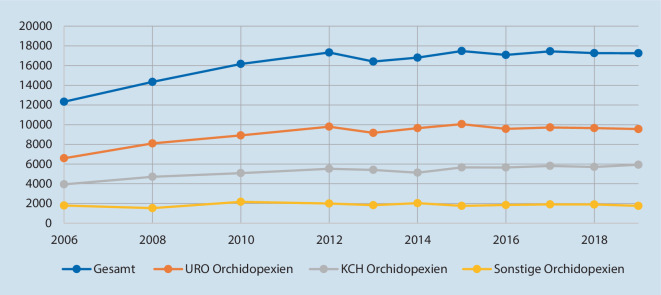

Die Abb. 1, 2 und 3 zeigen die Fallzahlentwicklung für die drei gewählten Indexeingriffe.

**Figure 2:**
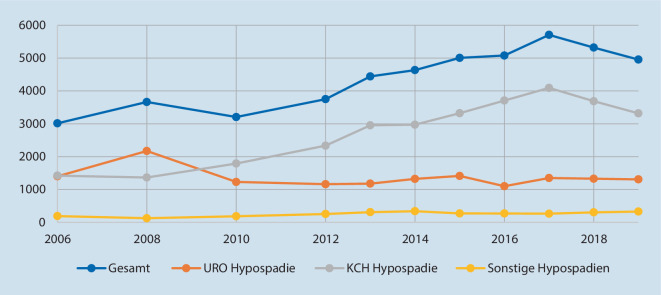

**Figure 3:**
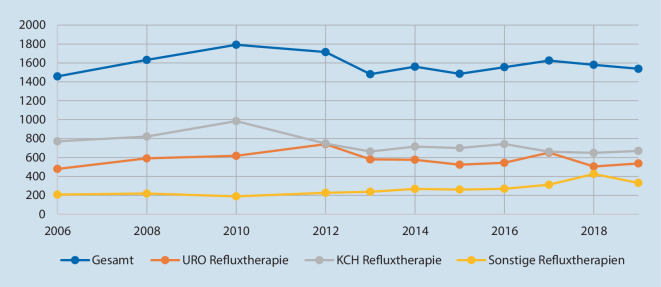

Von 2006 bis 2012 zeigte sich ein Anstieg der Orchidopexien um 41 % von 12.328 auf 17.322 Fälle pro Jahr (Abb. 1). Ab 2012 bildet sich ein Plateau mit konstanter Fallzahl aus. Sowohl die Gesamtzahl aller Orchidopexien als auch die Fallzahl in beiden Fachabteilungen steigt im Studienzeitraum an (*p* ≤ 0,01 für die Trends). Der Trend unterscheidet sich nicht zwischen urologischen und kinderchirurgischen Abteilungen (*p* = 0,18). Der urologische Anteil zeigt sich im Studienzeitraum durchgängig höher als der kinderchirurgische. Die Untergruppe der laparoskopischen Hodensuchen an allen Orchidopexien im Jahre 2019 beträgt 2,5 % (430/17.247), wobei der Anteil in der Urologie mit 1 % (90/9656) bei 36 durchführenden Kliniken deutlich geringer ist als in der Kinderchirurgie mit 6 % (340/5834) bei 61 durchführenden Kliniken (*p* < 0,001).

Über den Studienzeitraum steigt die Anzahl der Hypospadiekorrekturen um 64 % von 3015 auf 4955 Fälle (*p* < 0,001 für den Trend; Abb. 2). Während sich die urologischen Fälle nicht verändern (*p* = 0,2 für den Trend) findet sich in der Kinderchirurgie eine Zunahme (*p* < 0,0001 für den Trend). Zu Beginn des Studienzeitraums waren die Anteile an der Versorgung nahezu identisch, während die Kinderchirurgie seit 2010 höhere jährliche Fallzahlen verzeichnet als die Urologie. Damit unterscheidet sich die Entwicklung zwischen urologischen und kinderchirurgischen Abteilungen (*p* < 0,0001) im Hinblick auf diese Prozedur.

Von 2006 bis 2010 zeigt sich ein kontinuierlicher Anstieg der Gesamtzahlen der VUR-Therapien um 23 % von 1458 auf 1792 (*p* < 0,001 für den Trend; Abb. 3). Anschließend gehen die Fallzahlen bis 2013 zurück (*p* < 0,001 für den Trend) und verlaufen danach auf dem Niveau von 2006 seitwärts. In der Urologie findet sich keine Veränderung der Fallzahlen (*p* = 0,9 für den Trend) während sich in der Kinderchirurgie eine leichte Abnahme zeigt (*p* = 0,03 für den Trend); letztere erscheint aufgrund der niedrigen absoluten Fallzahldifferenz trotz des signifikanten Trends nicht als relevant. Dies gilt damit auch für den formalen Unterschied im Vergleich der beiden Fachabteilungen (*p* = 0,04). Der urologische Anteil zeigt sich im Studienzeitraum durchgängig niedriger als der kinderchirurgische.

### Zentrenbildung gemessen an den Fallzahlen von 2019

Die folgenden Analysen beziehen sich auf das Jahr 2019. Hier konnten wir aus der Gruppe der pädiatrisch entlassenen PatientInnen den operativen Fachabteilungen einen relevanten Anteil der Fälle zuordnen: Orchidopexie (35 % URO, 48 % KCH, 17 % unklar), Hypospadiekorrekturen (12 % URO, 34 % KCH, 54 % unklar) und Therapie des VUR (20 % URO, 39 % KCH, 61 % unklar). Diese Korrektur ist in den folgenden Daten enthalten.

Im Jahr 2019 erfolgten 9556 Orchidopexien in 427 urologischen Fachabteilungen (Mittelwert 22 und IQR 5–31 Fälle/Abteilung) und 5934 Orchidopexien in 107 kinderchirurgischen Fachabteilungen (Mittelwert 55 und IQR 24–69 Fälle/Abteilung). Für die Hypospadiekorrektur waren es 1308 Prozeduren in 127 urologischen Fachabteilungen (Mittelwert 10 und IQR 1–10 Fälle/Abteilung) und 3319 Prozeduren in 80 kinderchirurgischen Fachabteilungen (Mittelwert 41 und IQR 7–31 Fälle/Abteilung). Für die Therapie des VUR waren es 537 Prozeduren in 151 urologischen Fachabteilungen (Mittelwert 4 und IQR 1–4 Fälle/Abteilung) und 670 Prozeduren in 70 kinderchirurgischen Fachabteilungen (Mittelwert 10 und IQR 1–13 Fälle/Abteilung). Für alle drei Indexeingriffe ist damit das Abteilungsvolumen in der Kinderchirurgie höher (jeweils *p* < 0,001).

**Figure 4:**
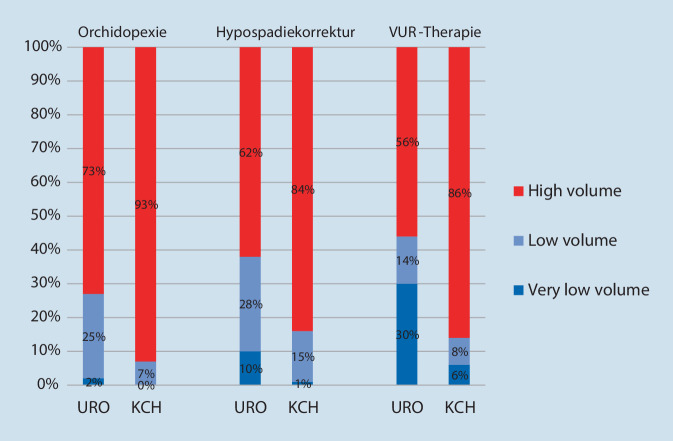

Die Abb. 4 stellt die Verteilung der Eingriffe auf die verschiedenen Fallzahlkategorien dar, wobei die High-volume-Kliniken für die Orchidopexie und die Hypospadiekorrektur mit ≥ 25 Fälle/Jahr definiert sind und für die Therapie des VUR mit ≥ 7 Fällen/Jahr. Als „very low volume“ haben wir < 5 Fälle/Jahr definiert, wodurch sich die Kategorie „low volume“ zu 5–24 Fälle/Jahr (Orchidopexie und Hypospadiekorrektur) bzw. 5–6 Fälle/Jahr (Therapie des VUR) ergibt. Der Anteil der Fälle die in High-volume-Abteilungen versorgt werden ist für alle drei Indexeingriffe in der Kinderchirurgie höher als in der Urologie, d. h. für die Orchidopexie URO 73 % vs. 93 % KCH, für die Hypospadiekorrektur URO 62 % vs. 84 % KCH und für die VUR-Therapie URO 56 % vs. 86 % KCH (jeweils *p* < 0,001).

Exemplarisch soll diese unterschiedliche Verteilung für die Hypospadiekorrektur im Jahr 2019 ausgeführt werden: In der Urologie gibt es 14 Fachabteilungen, welche mindestens 25 (bis maximal 170) Hypospadiekorrekturen durchführen. Diese stellen 62 % (807/1308) aller urologischen Prozeduren dar. In der Kinderchirurgie sind es 26 Fachabteilungen mit mindestens 25 (bis maximal 1025) Hypospadiekorrekturen, welche einen Anteil von 84 % (2787/3319) aller kinderchirurgischen Prozeduren ausmachen.

**Figure 5:**
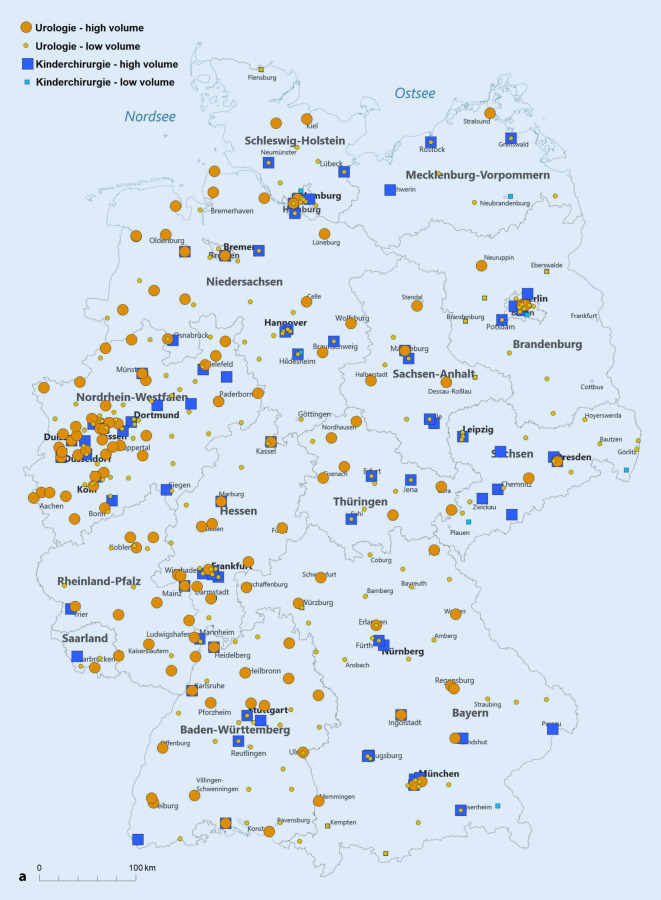

Zur besseren regionalen Einordnung der Versorgungssituation stellen wir die Abteilungen mit „high“ und „low volume“ (Abb. 5a–c) sowie die Bevölkerungsdichte (Abb. 5d) auf der Deutschlandkarte dar. Hierbei zeigt sich, dass die Verteilung dieser Abteilungen im Wesentlichen der Bevölkerungsverteilung folgt. Außerdem ist in den neuen Bundesländern die Kinderchirurgie traditionell stärker vertreten.

**Figure 6:**
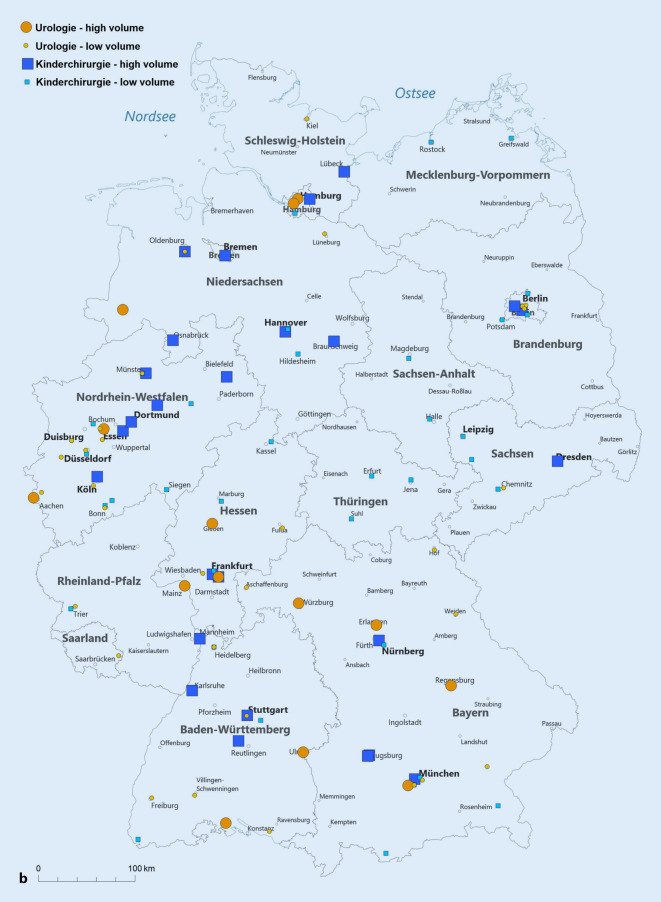

**Figure 7:**
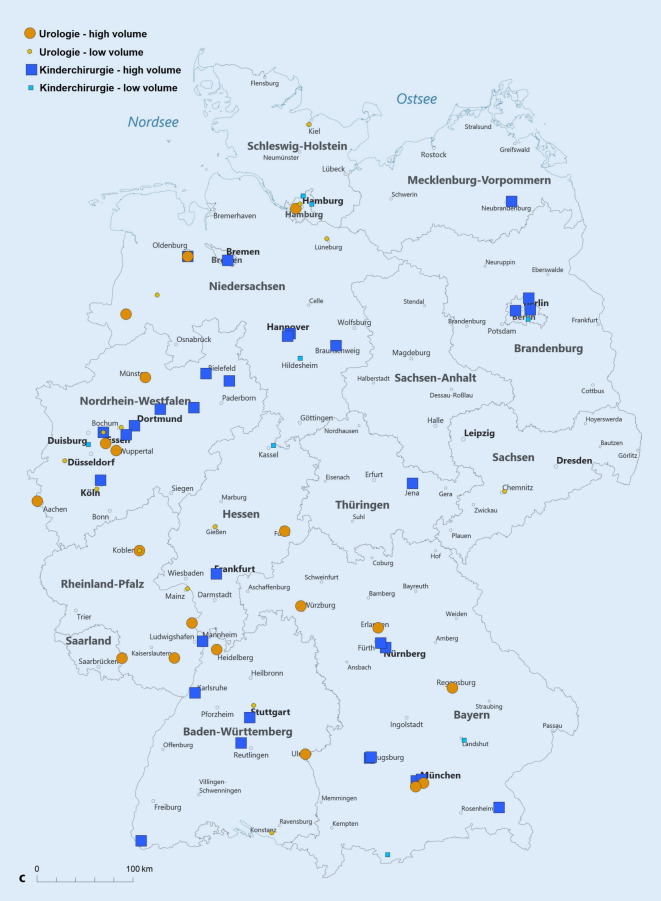

**Figure 8:**
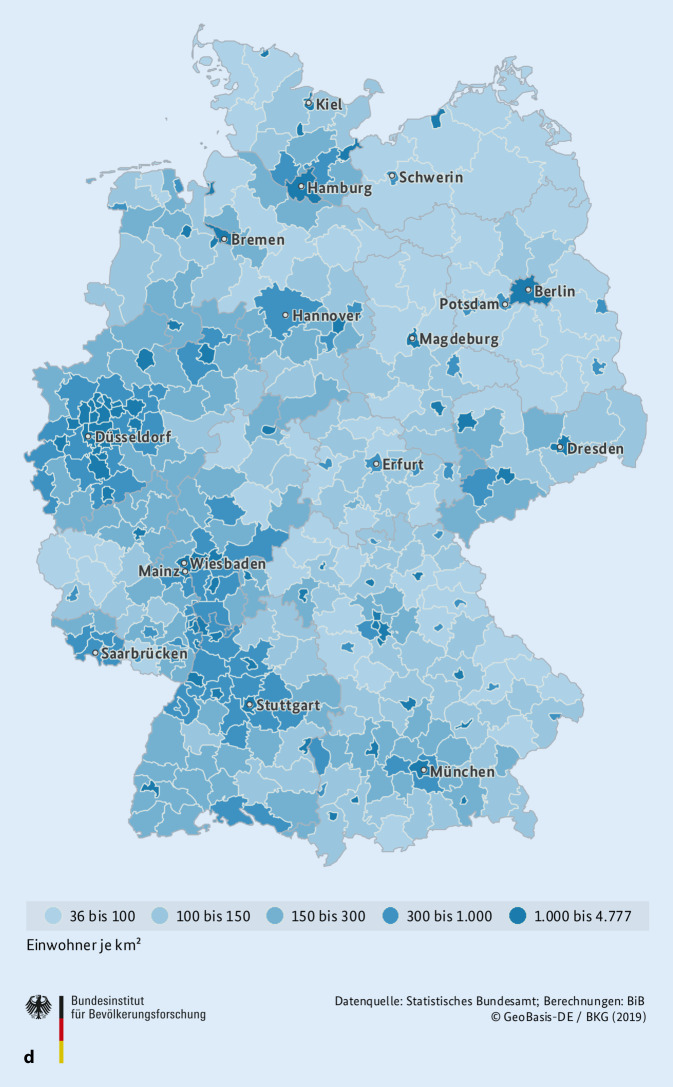

## Diskussion

Die drei gewählten Indexeingriffe werden von urologischen, explizit kinderurologischen und kinderchirurgischen Abteilungen durchgeführt. Der am häufigsten durchgeführte Eingriff, die Orchidopexie, erfolgt zahlenmäßig häufiger in der Urologie während die Hypospadiekorrektur und die operative Therapie des VUR häufiger in der Kinderchirurgie stattfinden. Für die prozentualen Fallzahlanteile der beiden Fachbereiche Urologie und Kinderchirurgie zeigt sich für die Orchidopexie und die Therapie des VUR keine relevante Verschiebung im Studienzeitraum. Bei den Hypospadiekorrekturen nimmt der Anteil der in Kinderchirurgien operierten Kinder seit 2006 zu (*p* < 0,0001). In der Kinderchirurgie erfolgen 84–93 % der Eingriffe in High-volume-Abteilungen während dieser Anteil in der Urologie nur bei 56–73 % liegt. Insbesondere die Refluxtherapie in der Urologie erfolgt zu einem hohen Anteil als Gelegenheitseingriff (30 % „very low volume“, d. h. < 5 Fälle/Jahr).

### Welche Einflussfaktoren nehmen auf die Fallzahlentwicklung Einfluss?

Insgesamt nahmen im Beobachtungszeitraum die Orchidopexien um 3,5 %/Jahr und die Hypospadiekorrekturen um 5 %/Jahr zu, während die Geburtenrate nur um 0,6 %/Jahr anstieg [3]. Rein demographisch lässt sich der Fallzahlanstieg also nicht erklären. Grundsätzlich ist die Fallzahlentwicklung von vielen möglichen Einflussfaktoren abhängig. In Qualitätsberichten werden weder historisch gewachsenen Standpunkte bestimmter Fachrichtungen begründet noch lokoregionale Besonderheiten, wie eigenständige kinderurologische Zentren adäquat abgebildet. Zu wenig ist über die Rolle der zuweisenden Kinder- und HausärztInnen und deren Beweggründe für die Einweisung bekannt. Neben persönlichen und fachlichen Erfahrungswerten spielen sicher auch Sympathien, Loyalitäten zu ehemaligen Ausbildungsstätten oder Fortbildungs- oder sonstige Serviceleistungen der Kliniken eine Rolle. Zudem ist weitgehend unklar, wie Eltern heutzutage medizinische Entscheidungen treffen [4]. Sicherlich werden die Eltern heute neben dem Rat der KinderärztIn in den Medien, Klinikwebseiten, Bewertungsportalen im Internet und über Mundpropaganda Informationen einholen. Die gemeinsame Entscheidungsfindung zwischen Eltern und Arzt, die auf fachlicher Kompetenz und Empathie fußt, sowie die operative Expertise sind entscheidend für die Auswahl des Krankenhauses an denen Eltern Ihre Kinder operieren lassen [5]. Zudem haben vor allem bei seltenen Erkrankungen die Selbsthilfegruppen eine entscheidende beratende Rolle.

### Ist Versorgungs- und Ausbildungsqualität an der Fallzahl messbar?

Im Allgemeinen gilt die Orchidopexie, die im frühen Alter vor dem 12. Lebensmonat empfohlen wird [6], als Eingriff der niedrigeren Schwierigkeitsstufe und ist auch in den Fertigkeiten des urologischen und kinderchirurgischen Facharztcurriculums enthalten. Die detektierte Fallzahlentwicklung seit 2006 lässt auf eine allgemeine adäquate Ausbildungsoperation hoffen, zumindest in der Kinderchirurgie, wo fast alle Kliniken (93 %) eine hohe Anzahl von Orchidopexien realisieren. Bei der Analyse zeigte sich aber auch, dass 73 % der urologischen Abteilungen die Orchidopexie in höherer Fallzahl durchführen (≥ 25 pro Jahr). Flächendeckend bieten 88 % der urologischen Abteilungen in Deutschland die Orchidopexie an (428/489 Fachabteilungen). Das Alter bei Operation, dabei vor allem der leitliniengerechte Zeitpunkt bis zum ersten Geburtstag, der bilaterale Hodenhochstand, die Hodenausgangslage und die chirurgische Expertise werden als potenzielle Outcomeprädiktoren diskutiert [7, 8]. Outcomedaten nach Orchidopexie in den hier als „low“ bzw. „high volume“ definierten Zentren Deutschlands stehen im Augenblick noch nicht zur Verfügung, sodass hier keine Aussage über die aktuelle Behandlungsqualität oder notwendige Mindestfallzahlen getroffen werden kann. Eine Norwegische Studie stellte die operative Expertise in Korrelation zu den Operationsergebnissen [9]. Zwar war grundsätzlich keine statistische Signifikanz der Ergebnisse von Chirurgen mit hoher Fallzahl (zwischen 18–55 Operationen) und Fellows (1–10 Operationen) zu finden. Erfahrene Operateure jedoch führten 82 % der Operationen mit 85 % Erfolg durch (Individualerfolg 71–94 %). Fellows mit bis zu 10 Eingriffen hingegen erreichten jedoch nur eine Erfolgsrate von 65 % (*p* = 0,02; [9]).

Eine entsprechend zu fordernde Fallzahl pro Operateur und/oder Klinik ist aber daraus schwer ableitbar. Die vorliegenden Qualitätsberichtsdaten erlauben leider auch dahingehend keine Rückschlüsse, da keine Komplikationen oder Outcomeparameter erhoben wurden. Für komplexe uroonkologische Eingriffe wie die radikale Prostatektomie [10–12] und die radikale Zystektomie [13, 14] ist der Zusammenhang zwischen Fallzahlen und Ergebnisqualität sehr gut belegt. Hier sind die Outcomeparameter klar definiert und standardisiert messbar. Dennoch existiert interessanterweise für urologische Indikationen in Deutschland nur für die Nierentransplantation eine Mindestmengenvorgabe [15].

Besonders für sehr seltene Eingriffe oder bei fehlenden gut operationalisierbaren Ergebniskriterien ist die Ableitung einer Mindestexpertise deutlich schwieriger [16, 17]. Dies trifft auch auf die meisten kinderurologischen Eingriffe zu. Wie Thorup et al. bereits 2011 schlussfolgerten, bleibt auch hier nur die allgemeine Aussage, dass z. B. Orchidopexien von „dedicated“ (hingebungsvollen, engagierten) Kinderurologen und Kinderchirurgen durchgeführt werden sollten, um das Komplikationsrisiko zu minimieren [18].

Der Anteil der Kliniken, die laparoskopische Verfahren in der Versorgung von Abdominalhoden einsetzen ist für die Urologie anteilig deutlich geringer als der Anteil in der Kinderchirurgie. Von Abteilungen mit Orchidopexien führen nur 36 von 428 (8 %) urologischen, aber 61 von 108 (56 %) kinderchirurgischen Abteilungen die Laparoskopie beim Abdominalhoden durch. Durchschnittlich (Mittelwert) werden pro Jahr in urologischen Abteilungen, die diese Maßnahme anbieten, 2,7 (IQR 1–2) laparoskopische Hodensuchen durchgeführt, wohingegen in kinderchirurgischen Kliniken 5,4 (IQR 1–8) derartige Eingriffe pro Jahr stattfinden. Neben einer Selektion anspruchsvollerer Fälle kann auch eine an der Expertise orientierte Operationsstrategie vermutet werden. In Metaanalysen zeigte sich das Outcome nach laparoskopischen Orchidopexien den Ergebnissen nach offen operativen Verfahren durchaus äquivalent [19]. Kinderchirurgische Kliniken haben jedoch im Alltag – nicht zuletzt durch die standardmäßig laparoskopisch durchgeführten Appendektomie – eine breitere laparoskopische Expertise am Kind. Auf jeden Fall kann aus den Zahlen geschlossen werden, dass die laparoskopische Behandlung des Abdominalhodens aktuell in der Urologie in wenigen Zentren in geringer Fallzahl durchgeführt wird.

Eurocat-Daten zeigen, dass die Hypospadie mit 18,6 (5,1–36,8) pro 10.000 Geburten eine häufige kinderurologische Anomalie in Europa ist [20]. Eine klare Zunahme der Krankheitsinzidenz in Europa konnte in epidemiologischen Studien nicht bewiesen werden [20]. Die in der Erhebung beobachtete operative Fallzahlzunahme kann auf zunehmend geplante und an der Pathophysiologie orientierte mehrzeitige Operationsverfahren, zunehmende Komplikationen und auf höhere kosmetische und funktionelle Ansprüche zurückzuführen sein. Auszuschließen ist anhand der vorliegenden Qualitätsberichtsdaten keinesfalls, dass die Fallzahlzunahme auch durch notwendige Rezidiveingriffe oder Eingriffe bei Komplikationen bei demselben Kind zustande kommen können. Dies ist durchaus plausibel, denn die operative Korrektur der Hypospadie ist besonders diffizil. In einer Metaanalyse zeigte sich die Rate der Komplikationen in Hinblick auf Meatusstenosen, Urethralstrikturen, Fisteln und Reoperationsrate abhängig von der Meatuslokalisation, der Primär- oder Sekundärrekonstruktion sowie der operativen Erfahrung des Behandlers [21]. Wie bei jeder Operation sind die Lernkurve und die generelle operative Erfahrung sowie die tägliche Routine (Fallzahlen) für das Outcome wesentlich [21–23]. So sinkt die Komplikationsrate mit den operativen Jahren und der Anzahl der Hypospadiekorrekturen in der Ausbildung linear [24]. In einer britischen Analyse, die als High-volume-Zentrum eine Operationsanzahl von 20/Jahr klassifizierte, zeigte sich die Komplikationsrate signifikant niedriger als in sog. Low-volume-Zentren (17,5 % vs. 25 %, *p* = 0,01; [23]). In einer populationsbezogenen US-amerikanischen Studie sank die Wahrscheinlichkeit einer größeren Revisionsoperation um 29 % pro 10 zusätzlich behandelten Hypospadien pro Jahr [22]. Generell fand sich im Untersuchungszeitraum eine signifikante Verschiebung der Hypospadiekorrekturen in die Kinderchirurgie; die Ursachen dafür bleiben in dieser Analyse unklar. Gerade bei der Indikationsstellung zur Hypospadiekorrektur darf jedoch das „Decisional-regret-Phänomen“ nicht vergessen werden, welches in bis zu 62 % durch unterschiedliche eltern- und patientenbezogene Einflussfaktoren und vor allem postoperative Komplikationen verursacht wird [25]. Der Einfluss operateurbezogener Fallzahlen bzw. der Versorgungsqualität auf dieses Phänomen ist bis dato unklar.

### Einfluss von geändertem Krankheitsverständnis auf die Fallzahl?

Im analysierten Zeitraum 2006–2019 veränderte sich das Verständnis der Refluxerkrankung und damit auch deren Diagnostik und Therapie signifikant [26, 27]. Seitdem versuchen die Kliniker nur noch einen „klinisch bedeutsamen“ Reflux zu finden und die Therapie mehr an der Pathogenese und den prognosegebenden Komorbiditäten zu orientieren. Eine risikoadaptierte Betrachtung in Hinblick auf den oberen Harntrakt und Komplikationen setzte sich als Grundprinzip durch [28, 29]. Beeindruckend sank die Zahl der durchgeführten Miktionszysturethrographien. Dabei wurden bei anteilig gleich hoher Refluxdetektionsrate mehr dilatierende VUR gefunden [29, 30]. Serien aus den USA dokumentieren zudem einen relevanten Rückgang der Gesamtzahl an Refluxoperationen, v. a. aber der endoskopischen Refluxtherapien [29, 31]. Dieser Trend zeigte sich auch in den deutschen Daten nach dem Jahr 2010 (*p* < 0,001). Um die Güte einer operativen Maßnahme im Rahmen der Refluxtherapie beurteilen zu können, müssten Outcomeparameter wie De-novo-Nierennarben, operative Komplikationen oder Refluxpersistenz oder -rezidive, der Anteil der urotherapeutischen Beratungen und die Elternzufriedenheit zugrunde gelegt werden. Hier ist die Anzahl der Refluxoperationen allein keinesfalls ein Gütekriterium für die angebotene Behandlung der Refluxerkrankung!

### Konsequenzen für die Zukunft

Die zitierte Stellungnahme der urologischen Fachgesellschaft fordert auch und insbesondere eine ausreichende operative Expertise mit höchster Versorgungsqualität [1]. Als Konsequenz daraus ist neben dem strukturierten Weiterbildungskonzept der Zusatzweiterbildung „Spezielle Kinder- und Jugendurologie“ eine an der Onkologie angelehnte Zertifizierung mit Qualitätskriterien für alle Kliniken sinnvoll, die Kinder mit kinderurologischen Erkrankungen versorgen. Derartige pädiatrische Zertifizierungen sind schon etabliert wie „Ausgezeichnet für Kinder“ [32]. Zertifizierungen im Europäischen Kontext haben neben Leistungskennzahlen über mehrere Jahre vor allem Strukturkriterien wie vorgehaltene Ausstattung, nachvollziehbare organisatorische Abteilungs- und Krankenhausgrundsätze, bereits vorhandene Zertifizierungen, personelle Ausstattung und Strukturen einer etablierten interdisziplinären Zusammenarbeit für die Bewertung zugrunde gelegt. Wie in der Stellungnahme gefordert [1], ist qualifiziertes Personal vorzuhalten (mindestens zwei Fachärzte mit der Weiterbildung „Spezielle Kinder- und Jugendurologie“), um eine Versorgung mit hoher Qualität bei komplexen kinderurologischen Eingriffen, aber auch in der konservativen Betreuung dieser Patienten gewährleisten zu können. Dies macht hier auch deutlich mehr Sinn als reine Fallzahlen, die u. a. auch von regionalen Umständen, oder auch übergeordneten Prinzipien wie der Leitlinienentwicklung oder berufs- oder krankenhauspolitischen Veränderungen abhängen können. Zudem könnte – und dies ist ein denkbar problematischer Ansatz – eine ausschließliche Fallzahlbetrachtung auch zu einer „tendenzielleren“ Indikationsstellung führen. Gerade die gegenüber uroonkologischen Eingriffen deutlich geringere Fallzahl in der Kinderurologie erfordert zudem ein strukturiertes Ausbildungskonzept, um auch zukünftig eine qualitativ hochwertige Versorgung garantieren zu können.

Die vorliegende Arbeit liefert eine orientierende Grundlage für die notwendige Diskussion zur sinnvollen Strukturierung der kinderurologischen Versorgung in Deutschland. Ein weiterer Vorteil der erhobenen Daten ist die regionale und transparente Zuordnung durch die verbindlichen Qualitätsberichtdaten. Eine bereits geplante Folgearbeit soll durch die Kombinationen aus Diagnose und OPS sowie dem Patientenalter eine detailliertere Analyse ermöglichen und die hier gefunden Daten und Aussagen vertiefen. Zudem macht die Analyse weiterer Indexeingriffe wie der Pyeloplastik Sinn. Mit zusätzlichen Daten aus einer DRG-Datenbankabfrage können auch Surrogatparameter für die Behandlungsqualität geprüft werden (z. B. das Alter zum Zeitpunkt der Orchidopexie), um ein umfassenderes Bild zu erhalten. Aufgrund der dann verfügbaren detaillierteren Daten wäre zudem zu hinterfragen, ob das flächendeckende Anbieten von kinderurologischen Eingriffen in fast jeder urologischen Fachabteilung geeignet ist, die urologische Expertise zur Behandlung von Kindern langfristig zu erhalten. Alternative Wege bis hin zur regionalen Abstimmung kinderurologischer Leistungen an ausgewählten Standorten oder fach- und standortübergreifende Kooperationen könnten erwogen werden, um die Ausbildungsqualität zukünftiger UrologInnen und die Behandlungsqualität der Kinder in Deutschland zu verbessern.

### Limitationen und Stärken

Diese Analyse der Qualitätsberichtsdaten hat eine Reihe von Limitationen. Die vorliegenden Daten hängen direkt von der Verschlüsselungsqualität ab. Abrechnungsformalien führen möglicherwiese an einigen Standorten dazu, von UrologInnen versorgte kinderurologische Fälle unter einer kinderchirurgischen Stationsbetreuung abzurechnen. Dies trifft zum Beispiel für das urologisch geführte interdisziplinäre Zentrum für Kinder‑, Jugend- und rekonstruktive Urologie am Universitätsklinikum Mannheim zu. Diese formalen Festlegungen führen zum Risiko einer Verzerrung mit Blick auf die Versorgungsrealität. Die aktuelle Analyse erlaubte keine Einschränkung für das Patientenalter, sodass nicht nur Prozeduren bei Kindern enthalten sind. Zudem war eine Kombination mit Diagnosecodes (ICD) nicht möglich, was eine gewisse Unschärfe insbesondere in Hinblick auf die Vollständigkeit und die Bewertung der Krankheitsschwere hineinbringt. Da die gewählten Prozeduren anderseits recht spezifisch für die entsprechenden Krankheitsbilder sind, ist für einen ersten Eindruck die Kombination mit dem Diagnosecode zunächst entbehrlich. Einzelbetrachtungen von Kliniken haben jedoch gezeigt, dass durchaus aufgrund der Verschlüsselung und der dort gewählten OPS-Codes erhebliche Fallzahldifferenzen auftreten können. Eingriffe im niedergelassenen Bereich sind in der Datengrundlage nicht enthalten, wobei dies nur für die Orchidopexie und manche milde Formen der Hypospadie wirklich relevant sein dürfte. Die Fallzahlfestlegung zur Einteilung der Kliniken wurde mathematisch (Mittelwert) aus den gegebenen Daten abgeleitet. Die formal eingegangene Wertung der Kliniken in high oder Low-volume-Zentrum ist somit primär deskriptiv, da kaum verwendbare Daten für Mindestmengen für die analysierten Eingriffe bezogen auf das operative Outcome vorliegen. Zudem müsste nicht nur pro Klinik sondern auch pro Operateur eine Aussage getroffen werden.

## Fazit für die Praxis

- Die Versorgungswirklichkeit der kinderurologischen Indexeingriffe wurde erstmalig anhand von Analysen der publizierten Qualitätsberichtsdaten für Deutschland durchgeführt.
- Kinderurologische Indexeingriffe werden seit Jahren konstant von urologischen und kinderchirurgischen Kliniken durchgeführt.
- Nur bei der Hypospadiekorrektur zeigt sich eine relevante Verschiebung in Richtung der Kinderchirurgie.
- Weitere detailliertere Analysen sind notwendig, um sinnvolle Rückschlüsse ziehen zu können.
- Erklärtes Ziel bleibt es weiterhin, die Ausbildungsqualität in der Kinderurologie als Teilgebiet der Urologie zu erhalten und die operative und konservative Versorgung der betreuten Kinder nachhaltig zu sichern.
